# How to establish and sustain a disease registry: insights from a qualitative study of six disease registries in the UK

**DOI:** 10.1186/s12911-024-02775-x

**Published:** 2024-11-28

**Authors:** Edmund Stubbs, Josephine Exley, Raphael Wittenberg, Nicholas Mays

**Affiliations:** 1 Policy Innovation and Evaluation Research Unit, Care Policy and Evaluation Centre, London School of Economics and Political Science, Houghton Street, London, WC2A 2AE UK; 2 Department of Health Services Research and Policy, Policy Innovation and Evaluation Research Unit, London School of Hygiene & Tropical Medicine, 15-17 Tavistock Place, London, WC1H 9SH UK

**Keywords:** Disease registry, Chronic conditions, Health research data and infrastructure, Patient registry, Long COVID-19

## Abstract

**Background:**

The advent of new chronic conditions such as long COVID-19 raises the question of whether and, if so, how best to establish new disease registries for such conditions. Prompted by the potential need for a long COVID-19 registry, we examined experiences of existing UK disease registries to understand barriers and enablers to establishing and sustaining a register, and how these have changed over time.

**Methods:**

We undertook semi-structured interviews between November 2022 and April 2023 with individuals representing six disease registries that collect individual-level longitudinal data on people diagnosed with a chronic condition.

**Results:**

Registries examined were developed by a few individuals, usually clinicians, to gain a greater understanding of the disease. Patient voices were largely absent from initial agenda setting processes, but, over time, all registries sought to increase patient involvement.

Securing long-term funding was cited as the biggest challenge; due to limited funds, one of the registries examined no longer actively recruits patients. Charities devoted to the diseases in question were key funders, though most registries also sought commercial opportunities. Inclusion on the NIHR Clinical Research Network Portfolio was also considered a vital resource to support recruitment and follow-up of participants.

All registries have sought to minimise the primary data collected to reduce the burden on clinicians and patients, increasingly relying on linkage to other data sources. Several registries have developed consent procedures that enable participants to be contacted for additional data collection. In some cases, the initial patient consent and data sharing permissions obtained had limited the flexibility to adapt the registry to changing data needs. Finally, there was a need to foster buy-in from the community of patients and clinicians who provide and/or use the data.

**Conclusion:**

We identified six key considerations when establishing a sustainable disease registry: (1) include a diverse set of stakeholders; (2) involve patients at every stage; (3) collect a core data set for all participants; (4) ensure the data system is flexible and interoperable with the wider data landscape; (5) anticipate changing data needs over time; and (6) identify financial opportunities to sustain the registry’s activities for the long term.

## Background

There is a long tradition of establishing patient registries to provide high quality real-world data to understand a disease’s (or multiple diseases’) natural history, to assess treatment effectiveness and safety, as well as support clinical practice and inform patient care [1–5]. A patient registry is defined as “an organised system for the collection, storage, retrieval, analysis, and dissemination of information on individual persons who have either a particular disease, a condition (e.g., a risk factor) that predisposes to the occurrence of a health-related event, or prior exposure to substances (or circumstances) known or suspected to cause adverse health effects” [6]. Registries aim to collect and routinely update uniform data from a population with a common feature [7, 8]. As such, they provide a rich source of patient data to examine a specific population, often capturing data not collected in routine health information systems.

The emergence of long COVID (also referred to as post COVID-19 syndrome) during the COVID-19 pandemic has led a number of countries to either develop (New Zealand, England), or recommend governments develop (Wales, Australia), a registry for long COVID [9–15]. The case for establishing long COVID registries is driven by the very limited understanding of its causes, manifestations, natural history, effective management or impact on patients’ lives. However, limited guidance is available in the published literature to inform decision makers how they might best go about establishing and sustaining a disease registry.

The experience of previous disease registries for long term health conditions offers valuable insights into how to establish and sustain a disease registry. We examined the experience of six disease registries in the UK to understand the barriers and enablers they have faced in developing and sustaining themselves and how these have changed over time. We used this information to identify key considerations for a successful disease registry for the long term.

## Methods

### Disease and participant selection

We included disease registries in the UK that aim to collect individual-level longitudinal data on people diagnosed with a long-term chronic condition. We defined a registry as any ongoing process of individual, patient-level, data collection on those with, or suspected to have, the disease which is regularly updated after contact with the research team or after every consultation with medical services. There is no comprehensive list of disease registries in the UK; we therefore used Google to search for registries associated with chronic conditions. The candidate list of conditions comprised: arthritis and rheumatism; asthma; cancer; cardiovascular disease; complex regional pain syndrome (CRPS); cystic fibrosis; diabetes; inflammatory bowel disease (IBD) including Crohn's disease; chronic kidney disease; lung diseases (general); lupus; motor neuron disease (MND); myalgic encephalomyelitis or chronic fatigue syndrome (ME/CFS); multiple sclerosis (MS); rare diseases; rheumatoid conditions; sarcoidosis/Sjögren´s syndrome; and vasculitis. From the initial list, we identified 13 registries: BILAG Biologics Register (BILAG BR); British Society for Rheumatology for Rheumatoid Arthritis (BSRBR-RA); CRPS UK Clinical & Research Network; IBD Registry; Lung Disease Registry; MND Register; MS Register; Sentinel Stroke National Audit Programme; UK Cystic Fibrosis (CF) Registry; UK Juvenile-onset Systemic Lupus Erythematosus (JSLE) Study Group; UK Primary Sjögren's Syndrome Registry (UKPSSR); UK Renal Registry (UKRR); and UKIVAS Vasculitis Registry.

Key informants from each registry were purposively selected based on their close involvement in the development, management and/or running of these datasets. They were identified on the grounds that they would have sufficient familiarity with the datasets, the organisation, funding and current issues facing the registry to be able to respond to our questions. For each registry we aimed to identify a named individual and their personal email, although for two of the registries we were only able to identify a generic email address.

### Data collection

We approached potential key informants from each registry via email and provided them with background information on the purpose of the study and the consent form. Potential interviewees were also sent a list of topics that would be covered in the interview. The topic guide covered the following areas: aims and objectives of data collection; sampling and recruitment process; data collection and measurement; management and infrastructure; data governance; accomplishments and impact; funding; support from other organisations; and perceptions of the quality of the dataset. We offered to hold group interviews so all relevant individuals from a registry could contribute. Up to two reminder emails were sent. Participants provided written informed consent before taking part.

Interviews were conducted using the video platform, Zoom, between November 2022 and April 2023, and with consent, were recorded and transcribed verbatim. Interviews lasted 45 min to an hour.

### Data analysis

Data were analysed thematically [16, 17]. We developed a coding framework a priori based on the overarching areas covered in the topic guide. All interviews were coded against the framework in Excel by one researcher (JE or ES). Additionally, any data from literature shared by interviewees post-interview were also extracted into this framework. The populated coding frame was shared with interviewees via email for their review and to clarify any points of uncertainty that had arisen during the initial coding. Subsequently, the data contained within each code were summarised and key themes within and between codes were identified inductively. The key themes were finalised in discussion with all researchers in the study.

### Ethical approval

The project was approved on 16/09/2022 by the London School of Hygiene & Tropical Medicine's Research Ethics Committee (approval number: 28096).

## Results

Of the 13 registries approached, we were able to conduct interviews with representatives from six disease registries. We were unable to secure interviews with the remaining 7 registries approached. An overview of the history, aims and organisations involved in each registry is presented in Table 1. Since completing the interviews, the IBD Registry announced in January 2024 that is closing; the Royal College of Physicians will act as interim steward for safeguarding the data but no data processing will take place [18–20].

**Table 1:** History and current aims of the sampled registries

Registry	History/Development	Current aims	Organisations involved	Funding
British Society for Rheumatology Biologics Register for Rheumatoid Arthritis (BSRBR-RA) [21]	Launched in 2001, when anti-TNF therapies were first rolled out across the NHS. All UK consultant rheumatologists who prescribed anti-TNF, and other targeted therapies, were recommended to enrol patients by the British Society of Rheumatology (BSR) to monitor the safety profile of these drugs	To monitor the long-term safety of biological and other targeted therapies used to treat rheumatoid arthritis	• British Society for Rheumatology • The Biologic Studies Group at The University of Manchester • Pharmaceutical Industry	• British Society for Rheumatology, which receives funding from pharmaceutical companies
Complex regional pain syndrome (CRPS) UK Registry [22]	Established in 2008 by a network of clinicians, therapists and researchers with an interest in CRPS (CRPS UK Clinical & Research Network). There was interest to explore how best to capture a population suffering from CRPS and facilitate epidemiology studies and clinical trials	To raise awareness and understanding of Complex Regional Pain Syndrome amongst health professionals, patients, and the public	• CRPS UK Clinical & Research Network • Cambridge University Hospitals NHS Foundation Trust • Royal United Hospitals Bath NHS Foundation Trust	• Not directly funded; built funding into research studies to cover cost of an administrator
Inflammatory Bowel Disease (IBD) Registry [23]	Formed in 2012 by a group of consultant gastroenterologists to help provide a better picture of IBD in the UK The IBD Registry officially launched as an independent not-for-profit in 2018 Announced its closure in January 2024	To improve treatment and care for people with IBD, and support research	• Royal College of Physicians • British Society of Gastroenterology • Crohn’s & Colitis UK	• Variety of sources including grants from both public institutions and commercial entities
UK Juvenile-onset Systemic Lupus Erythematosus (JSLE) Cohort Study and Repository [24]	In 2006 a group of paediatric rheumatologists, nephrologists and other specialists formed the UK JSLE Study Group, in partnership with LUPUS UK The Group established the UK JSLE Cohort Study and Repository in 2006 to facilitate research, supported by LUPUS UK	To improve both patient care and improve understanding of the disease Develop a comprehensive research programme to investigate the clinical characteristics and immunopathology of JSLE	• UK JSLE Study Group • University of Liverpool • Alder Hey Children’s NHS Foundation Trust • LUPUS UK	• Variety of individual grants and charitable sources supporting core administrative support only, including at various times, LUPUS UK and Versus Arthritis
Multiple sclerosis (MS) Register [25]	Launched in 2011 by the Population Data Science team at Swansea University Medical School to capture real world data about people living with MS in the UK. Initially aimed to provide a better estimate of the number of people suffering from MS in the UK	Build the evidence needed to campaign for fair policies and improved healthcare Build a clearer picture of the true impact MS has on people’s lives Be a resource for people with MS to track, store, access and then share their own data with their healthcare provider	• Swansea University Medical School • MS Society • Currently in process of linking with registries in US, France, Switzerland & Sweden	• MS Society
UK Renal Registry (UKRR) [26]	Established in 1995 by the Renal Association to improve the care of patients with end-stage kidney disease. Originally limited to people on kidney replacement therapy. Has expanded to include cases of acute kidney disease	To record and analyse longitudinal health data about children and adults with kidney disease in the UK for audit, quality improvement and research purposes	• British Renal Association • UK Kidney Association	• Capitation fee per patient on renal replacement therapy paid by the NHS • UK Kidney Association

All registries we examined include individuals who had received a clinical diagnosis, see Table 2. In addition, the UK JSLE Cohort Study includes children and young people presenting at rheumatology/renal clinics who do not yet meet the full criteria for a lupus diagnosis to gain insight into whether, and who, goes on to develop lupus. Across the registries examined, participants are either recruited when attending a specialist service (BSRBR-RA, JSLE, UKRR, CRPS), register themselves directly (IBD) or are recruited via both routes (MS); the CRPS Registry is no longer recruiting participants. The registries range in size from 618 patients enrolled in the CRPS Registry to 500,000 with acute kidney injury in the UKRR. Most datasets capture clinical data gathered during routine appointments with specialists in secondary care. The exception is the CRPS, in which data is only collected through an annual self-reported questionnaire. Only the MS Register is currently routinely collecting data on patient-reported outcome measures (PROMs).

**Table 2:** Recruitment and data collection summary

Registry	Eligibility criteria	Recruitment strategy	Consent	No. participants	Data collected	Linkage
*Patients attending specialist services*						
BSRBR-RA [21, 27]	• Aged 16 yrs or over • Clinical diagnosis of rheumatoid arthritis • Starting eligible biologic treatment	• Recruited from all NHS trusts that have a Rheumatology department when starting a new biological treatment either during visit to department or via an outreach consent • Requirement to recruit all eligible patients initially mandated by NICE, and now recommended by the British Society for Rheumatology • On the NIHR UK CRN Portfolio, means Centres in England have access to UK CRN nurse support/infrastructure support to help cover some of the costs of recruitment of participants	• Written informed consent to: 1. complete follow-up questionnaires about health; 2. for NHS hospital medical records to be shared with the study team; 3. for their NHS number to be shared with other national healthcare databases; 4. be contacted for other studies; and 5. pseudonymised data being shared with pharmaceutical companies for regulatory purposes only	• 30,000 patients from 100 + rheumatology clinics across the UK • Followed until withdraw from study or death	• Rheumatology department; data captured at baseline, then every 6 months for three years then once a year • Patient diaries & questionnaires (until 2019, when registry moved to an electronic system, currently on hold but an online patient PROMS system is in development). • During COVID-19 BSR developed an ePROMs tool to ensure data captured while not being seen routinely face to face (not currently linked to BSRBR-RA) • NICE mandated between (2003–2008) that all English patients on biologic drugs must have data submitted to the registry	• Collect NHS number to enable linkage with datasets held by NHS Digital^2^ e.g. cancer and death outcomes, Hospital Episode Statistics (HES) • Previously linked to Public Health England’s Myocardial Ischaemia National Audit Project (MINAP) • Exploring feasibility of linking to the BSR UK Juvenile Idiopathic Arthritis (JIA) Register
CRPS [22]	• Any age attending one of the approved NHS sites • Meet the diagnostic criteria developed by the CRPS UK Network (as there is not official diagnosis for CRPS), based on presenting symptoms and signs	• Approved staff from each of the 10 approved NHS sites were responsible for recruiting patients • Since December 2020 no longer recruiting new participants	• Written informed consent to provide personal contact details so can be contacted for: 1. contacted for follow-up; and 2. other studies	• 618 patients from 10 centres in England, Northern Ireland and the Republic of Ireland • No defined endpoint	• Baseline data collected by consenting clinician • Annual patient questionnaire	• No linkage
JSLE [24]	• Aged 18 yrs or under • Receiving care in a rheumatology or renal clinic in England or Scotland Two groups: 1. Meet international diagnostic classification criteria for lupus 2. Do not yet meet full criteria for a lupus diagnosis (“potentially evolving”)	• Recruited from the 23 children’s rheumatology clinics across England and Scotland	• Written informed parental consent / child assent given before inclusion in the Study, with individual consent for each component of the Study as appropriate	• 846 children in England and Scotland (no contributing centres yet listed for Wales and Northern Ireland) • Followed until discharged from care	• Rheumatology or Renal Department captures data at diagnosis and each subsequent visit • Annual assessment, which includes blood and urine specimens	• Many collaborative studies nationally and internationally, on-going
UKRR [26, 28–31]	• All patients being cared for by hospital kidney centres with chronic kidney disease, patients receiving dialysis and patients with a kidney transplant in England, Northern Ireland, Scotland^1^ and Wales • Expanded to include acute kidney injury in primary & secondary care in England & cases of advanced chronic kidney disease in secondary care, not on kidney replacement therapy, in England & Wales	• All NHS renal centres are required to submit data for patients meeting inclusion criteria, as set out in the NHS Renal Service Specification (A06). The Chief Executive of each trust is responsible for adhering to this contract	• Under Sect. 251 of the NHS Act 2006, data can be collected without consent for auditing purposes • Exempt from the National Data Opt-out programme; means patients cannot opt-out of sharing their data but can opt-out of sharing their identifiable data; this data cannot be linked to any other dataset • Patients can consent to being approached to participate in research	• Approx. 70,000 on kidney replacement therapy • 500,000 with acute kidney injury • Covers 71 adult & 13 paediatric renal centres in hospitals across England, Wales and Northern Ireland^1^ • Followed until death	• Renal centres must report data four times a year. Ongoing warehousing project (UK Renal Data Collaborative) aims to provide data in real time • Hospital laboratories • NHS England’s renal clinical reference group requires all centres to submit to the registry	• Collect NHS number to enable linkage e.g. with NHS Blood and Transplant, UK Health Security Agency data
*Patients sign up directly*						
IBD [32]	• Adults (although plans to expand to children & young people) • Confirmed diagnosis of Crohn’s disease, ulcerative colitis, IBD unclassified or another type of IBD • Living in the UK	• Self-sign up through an online form on the IBD Registry website. Must provide their NHS number and date of birth	• Patients consent to having their medical records shared with the registry • Due to a change in consent procedure in 2022 Registry consists of two datasets: 1. Original dataset supplied by NHS Digital^2^, only available as anonymised data 2. Personally identifiable data held on own data platform	• Over 85,000 records from 103 partner NHS hospitals in the UK (not including the Channel Islands and Isle of Man) • Followed until withdraw from study or death	• Gastroenterology unit captures data at each visit • Participants consent to being sent questionnaires, although not currently routinely done	• Original dataset stripped of identifiers so linkage not possible • Personally identifiable data contains NHS number and date of birth ability to link medical records with registry data
*Both*						
MS [33]	• Aged 16 yrs or over • Confirmed diagnosis of MS • Living in the UK • To provide clinical data must be receiving treatment from one of the NHS centres associated with the registry	Two routes: 1. Neurologists in large NHS hospital treatment centres associated with registry invite patients to participate when they are diagnosed 2. Individuals sign up directly through the website to participate in questionnaires	• Patients consent to having their medical records shared with the registry • Individuals who sign up to both elements can consent to have their clinical data and questionnaire data linked, and for their questionnaire results to be shared with their clinician	• Between 10,000–20,000 • Est. 5,500 engaged at each time point • Covers the UK, but no NHS partners recorded yet in Scotland • No defined endpoint	• MS clinical sites complete an electronic case report file every 6 months to a year • 6-mothly patient questionnaires	• Link to routine NHS data via SAIL Databank • Provide a data linkage services so researchers can link their own data to the registry

1 Scotland has its own Renal Registry (the Scottish Renal Registry), which is merged annually to provide coverage for the whole of the UK. 2 NHS Digital is now part of NHS England

*NICE* National Institute for Health and Care Excellence

We identified four key themes that were reported to have enabled or hindered the ability to sustain a disease registry. These were: (1) the need to anticipate how data will be used and how this might change over time; (2) unstable funding sources posing a threat to registries’ sustainability; (3) strategies to reduce the burden of primary data collection; and (4) the need to build patients’ and clinicians’ buy-in.

### *“Start with the end in mind"*: need to anticipate how data will be used and how this might change over time

One of the key lessons was the need to be *“really clear what your goals are”* from the outset, otherwise registries risk turning into a *"basket collecting data on all sorts of things with no specific question in the end that you can answer […] can have lots of questions, but unless the questions are clear you can't be sure you're collecting the right data to answer them"* (JLSE). The registries we examined were predominantly developed by clinicians and consequently, at least initially, the focus was on the data needed to support clinical knowledge. The patient voice was sometimes absent from the agenda setting process, but all interviewees considered that this reflected the norms at the time the registries were established. Interviewees discussed ongoing initiatives to increase patient engagement and involvement, such as UK JSLE which was developed with input from LUPUS UK and patients/families.

*“[Y]ou need to involve patients and the families, if it's involving children and young people you’re including, and that needs to be done in a very meaningful way. So not just a tick box at the end of the day, but really starting by speaking in meaningful ways to a cross-section of patients/families as well as representatives, you know groups of patients and families, and the public at large […]. So, you need to hear what the views of are around the questions that you've got, and listen to the questions that they have that they want you to answer as well”* (JSLE)

Linked to the need to broaden the scope and include a more diverse set of stakeholders, interviewees from the IBD and JSLE registries discussed the need to consider which stakeholders should be represented on the board or any steering committee and the value of including individuals from the different areas that the registries touch on.

*“The big reflection that's going on at the moment by the board themselves is, actually are we the best people? Should we have a more representative board. […] So, there is a piece of work on going to say, do we have the best board to govern what we do?”* (IBD)

Before undertaking any data collection, a number of interviewees highlighted the need to ensure there is a deep understanding of the wider data landscape. Interviewees from the CRPS and JLSE registries stressed that this understanding needed to extend beyond England to the rest of the UK to facilitate harmonisation of efforts initially within the UK but then internationally. One mechanism to support this was having representatives with knowledge of the wider data landscape on the steering committee.

*“[England is] only a drop in the world's research landscape […] so you need to be compliant with fields and elements that would be collected by […] all the nations who will be wanting to do a similar thing. I would urge international [collaboration], and that takes you into the realms of how you're collecting data, and what are the data sets you use and standardisation of data sets. […] the more your proposed study can plug into these, then it can become a central part of international collaborations and add to or even lead initiatives rather than being isolated in an island that no one else can connect to, even within the UK.”* (JSLE)

Data collection was deemed to be both costly and burdensome (see below). Consequently, we were told that registries should be seeking to draw on existing data collections wherever possible. All of the registries, except the CRPS, have the ability to link to other datasets; although a number of interviewees commented they had experienced considerable delays in access to data from NHS Digital (now part of NHS England). Alongside linkage, technological advances were also reported to offer opportunities. For example, the MS Register is exploring more innovative methods, such as machine learning, to extract data from existing sources.

*“[W]e try to apply natural language processing techniques to outpatient and inpatient letters, so we can harvest a lot of the details that we want from our population for those consented people, and that’s explicit in our consent form too”* (MS)

The data management system where the registry is housed was reported to play a critical role in the feasibility to undertake linkage. The interviewee from the MS Register discussed the value of storing the register in a trusted data environment. In the case of the MS Register, data are held in a Secure eResearch Platform (SeRP) at Swansea University housed within Wales’s SAIL (Secure Anonymised Information Linkage) Databank, which hosts a variety of linkable routine health datasets and other disease registries [33–35].

*“[T]he fact is, you know, all the right like security standards […] you've got the right governance in place, you've got the right method of accessing data in place, you've got the right screening to make sure that only the correct things go out […] we’re not hanging on to all of this silo data to ourselves”* (MS)

Other registries, such as UKRR and IBD Registry have developed in-house capacity to store and manage data. The IBD Registry moved from being held by NHS Digital to in-house in 2022. When the registry was started it was seen to be beneficial to use NHS Digital to provide a *“secure trusted name”,* but the data shared was anonymous which was reported to limit what could be achieved. The decision to move in-house was taken to give them “*more speed and flexibility”* and the data now comes with identifiers. At the end of March 2024 the IBD Registry was transferred to the Royal College of Physicians, who is acting as an interim steward while the registry finds a new home [18].

Developing a registry and its governance structures was reported to be a time-consuming process. However, investing resources upfront was considered a worthwhile to reduce the likelihood of having to make changes later.

*“I think of it [the registry] like the honeycomb. Hasn't got honey in it, but it took ages to build the honeycomb and get the information governance around it and get all these trusts to sign up"* (IBD)

Where changes were made, some were seen as unavoidable. For example, a number of registries were paper based when originally set up and have either moved or are in the process of moving to an electronic system. Other changes were seen as being more avoidable. For example, the original patient consents and data sharing permissions obtained were highlighted as placing limitations on what can be done with the data. Interviewees from the IBD discussed the inherent tension between wanting to be highly specific around how data would be used, and by whom, to foster trust and transparency, and the desire to be able to adapt systems over time. Interviewees urged that in setting up any new registry it would be vital to try to anticipate the different ways in which data needs might evolve over time and build in mechanisms that allow for some flexibility in data use.

*“I guess making any wording that you do, or any permissions that you get as broad as possible, so that when your scope changes slightly, which it will, the consent you gained originally still covers what you're trying to do now because we've been tripped up by that just a couple of times where we thought that in a patient information leaflet oh, we'd be, you know we're giving them lots of information[…]. But then, of course, when we changed that meant that all of the consents weren't valid anymore. So, it's just thinking how you can give enough information to them without pinning yourself down […]. We've got ethics approval for research database as opposed to specific studies. So, someone could come to us and apply for our data, and we could say, yes we're happy for you to have that and do that research.”* (IBD)

### *"It costs a lot to fund a good register”*: unstable funding posing a threat to sustainability

Interviewees told us that both establishing and maintaining a disease registry are expensive and that costs can rapidly escalate. Costs extend beyond *“databases and technical people, but it's almost as much in comms and engagement and reassurance and public work”* (IBD). Securing long term funding was therefore cited as one of the most challenging aspects to maintaining a registry. The CRPS Registry has stopped recruiting patients, although continues to collect data on existing participants, because of a lack of funds. The registry has survived through the commitment of the community of interested clinicians and researchers and *"a wing and a prayer and a nice piece of cake. It seriously is at that level".*

Initially the CRPS hoped the pharmaceutical industry would provide some funding but in *“2012 the entire pharma industry in the UK decided it was not going to fund nor research pain anymore, and that was a massive blow […] but these things go around in circles, and perhaps there is a bit more interest nowadays than there was. But subjected to these kind of trends […] they’re either flavour of the month or no one’s going to touch them with a barge pole”*. In contrast, the BSRBR-RA has been fully funded by the pharmaceutical industry since its inception in 2001 via the British Society of Rheumatology. This has proved a stable source of funding as the pharmaceutical industry is required by regulatory agencies to undertake post-market surveillance of new drugs prescribed for rheumatoid arthritis. Funding is currently in place until 2028, with further funding dependent on new products to treat rheumatoid arthritis being developed. Other registries have also found commercial opportunities to raise funds, for example, the IBD and MS registries undertake commissioned research for both public and private organisations.

Where data are used commercially, there is a need to build trust and transparency with patients and patient representatives. The interviewees from IBD argued that *“you cannot be open enough”* with patient groups. IBD is seeking to strengthen the role of its patient advisory group *“all the way through the organisation”*. In the case of the BSRBR-RA, the British Society of Rheumatology acts as an intermediary between the research team and the pharmaceutical companies providing the funding [36], although the interviewees considered that attitudes towards the pharmaceutical industry were changing.

*“[P]eople felt that academic research had to be separate from anything to do with the pharmaceutical industry. So BSR [British Society of Rheumatology] were kind of that sort of buffer between the two. You know, we're the independent scientific research group, and there's the pharma who are given the funding, but it's coming via the BSR, and the pharma have no play on the research that's coming out, and I think that was that was really important at the time. I don't think that's the case now. I think that there's very much more close links between pharma and Academia, and I think that's widely accepted now. So, in that sense, you probably don't need an umbrella organization like the BSR. But I do think it's still incredibly useful, whether it be a patient focused group, or whether it be a clinical focused group, or whether it be both, to get buy in."* (BSRBR-RA)

Charities devoted to the diseases in question were also cited as key sources of funding. Interviewees perceived that these organisations were incentivised to fund registries as they raise the profile of the condition, and the data provide an important advocacy tool for new treatments and to improve the care of the individuals they represent.

*“[T]hey continue to fund [the register] because I think beyond the intrinsic value, because we're expensive, [...] it's producing good research, you know, at the end of the day you put money in you want papers out. They're in higher quality journals, and they have more impact to the MS Society, so they can justify their investment."* (MS)

Beyond direct sources of funding, several interviewees discussed the value of access to NIHR Clinical Research Network (CRN) support, gained through their inclusion on the NIHR CRN Portfolio. This provides additional funding to support participating sites with recruitment and follow-up of patients, although not all registries have been able to secure this funding.

*“We got CRN support early on, and you know that was a hugely important thing […] if it is adopted then the infrastructure that’s in place in every NHS or an NHS setting will be additive to the research costs [..] it has to plug into NIHR infrastructure as much as you can”* (JSLE)

To reduce costs, interviewees highlighted the need to have a good understanding of the wider data landscape: *“I'm always looking for reuse and partnering rather than let's build it all again”* (IBD). Where primary data collection was necessary interviewees discussed the need to limit the amount of data collected to avoid escalating costs.

### *“You need to keep the dataset manageable so that you have good quality and complete data”*: strategies to reduce the burden of primary data collection

Tied to the financial costs of data collection, interviewees were also conscious of the burden data collection can place on both clinicians and patients. Where data collection was overly onerous, it was reported to harm the quality and completeness of the data. The pandemic and NHS staff shortages were perceived to have further reduced the ability to collect data.

*"[T]he NHS is so stretched, and I would say that that is one of the themes in terms of data quality, when we send back our queries and our summaries, it's getting trickier to get people to actually have the time to look at those queries"* (UKRR)

Interviewees discussed a number of strategies taken to reduce the amount of primary data collected, in particular moving to linkage to reduce pressure on sites.

*"There's only so much you can ask nurses and doctors to do without giving them any recompense. Wee need to be really aware of what we're asking, how much we're putting on the sites, and because they have so little time. So, it's so hard, and we're trying to move more to linkage rather than putting the pressure on the sites. And we've tried to strip down the questions that we're asking the sites as well."* (BSRBR-RA)

A number of the registries have streamlined the data items to a core set of measures, working with clinicians to build a consensus on what is most important to understand from a care point of view. In doing so, they have aimed to integrate data requests into routine care.

*“[T]he data collection is integrated into standard care, so that required a huge amount of engagement with clinicians across different specialities […] therefore you need to ensure that you have a very active engagement programme, also with patients/families””* (JSLE)

Interviewees from some registries pointed to challenges that the lack of a standardised IT system across the NHS posed to data collection. To reduce the burden on clinicians, the MS and IBD registries have developed *“system-agnostic”* tools. However, interviewees reported that data are submitted in different formats. As a result, a lot of data cleaning and validating is required before data can be uploaded to the registry. The UKRR employs a dedicated in-house team. While this was noted to save clinicians’ time and facilitate buy-in, it generates extra costs for the registry.

*“It would be tool-agnostic, was the phrase, because every trust has a different system […] There, wasn't a one size fits all. It would be system-agnostic. Here is a tool. If you want to use the tool. If you already got your tool, you could upload to us from your tool.”* (IBD)

The IT system was also reported to limit the flexibility to change the data collected.

*"[H]ave a well-established and published change management process, so that you, aren't adding data items willy nilly because it's difficult for centres to, if they're relying on a renal system supplier, then there's all the work that goes into adding data items on to the system, then, having it mapped within the hospital, and then making sure that people know that they're supposed to be recording that data and all those sorts of things.”* (UKRR)

Registries were seen to reduce the burden on patients by acting as a central resource, *“a one-stop shop […] [patients] don’t have to be putting their information everywhere all of the time”* (MS). Where data are lacking, many registries have developed mechanisms to expand the data collected either through data linkage or by approaching participants to participate in additional data collection. For example, during the COVID pandemic the BSRBR-RA, IBD, MS and UKRR registries all expanded data collection to capture the impacts of COVID-19. The ability to be able to either collect more data or contact participants is dependent on prior consent being sought.

*“We have NHS ethics to ask, you know, staff and patients and people with MS. This is flexible enough that we can put additional instruments as is required and we have in the past. It’s one of the services we offer for other researchers of MS and other conditions”* (MS)

### *“We recognise that there needs to be a carrot”*: maintaining patients’ and clinicians’ buy-in

Building and maintaining buy-in was reported to be challenging. For example, NHS hospitals were required to submit data to both the BSRBR-RA and the UKRR; for the BSRBR-RA, *“historically NICE actually mandated that all patients on these new biologic drugs should be registered and followed within this system. So we got really good buy in from the rheumatology community”* while participation in the UKRR is specified in the NHS Renal Service Specification (A06) and the chief executive of each Hospital Trust is responsible for ensuring data are recorded accurately on local IT systems and all required data are uploaded electronically to the UKRR [30]. However, in both cases, this has proved impossible to enforce and in 2008/2009 NICE *“decided not to mandate it anymore, and after that it was the British Society for Rheumatology that then put their recommendations in place that all patients should be registered on these drugs in the study”*. Interviewees linked this to the lack of a financial mechanism to make this happen.

*“So [NHS Renal Service Specification (A06)] sets out that renal centres must be able to send 100% of our dataset to us in electronic format. […] the problem I feel is that because […] not being part of the NHS, because we’re completely independent, but I think historically it’s been a problem because we don’t have any teeth. It hasn’t been enforced, it’s not like CQUINs [NHS Commissioning for Quality and Innovation incentive payments] where there is finance associated with whether you return the data. So, moving forward with the data warehousing and out new dataset we’re pretty sure that it is going to be mandated by NHS England. So, we’re just waiting for that to come out which will make life easier”* (UKRR)

In the absence of financial remuneration, interviewees across registries recognised the need to provide other incentives to foster buy-in. The involvement of a network of interested clinicians was seen as one mechanism to develop *“social buy-in and some sort of moral or ethical driver”*, while being able to demonstrate the impact data are having on the understanding of the condition, clinical practice and patient care was another. All registries publish routine insights and provide access to the data to support academic research.

*“[W]e have strong buy in. I mean we don't provide the centres with any payments or any reimbursement for all of this data but you know that there's so many clinical decisions made based on the data that's come out of the register.”* (BRSRB-RA)

However, the timeliness of the data was reported to limit the value to day-to-day decision making in clinical settings. To better support clinical decision making and provide more timely assessment of patient care, the UKRR is currently supporting renal centres to transition from the existing system, in which data are submitted on a quarterly basis, to receive daily updates from renal centres submitted via the UK Renal Data Collaboration [28, 37, 38].

*“When the registry was originally set up nothing like it had existed before, so the publication of an annual report was incredibly useful for comparison and audit but now things have moved on. And actually, by the time we’ve collected the data and published the data is, you know, two years old and people want data now”* (UKRR)

Improving access to the data was also seen as important to support patient engagement. Both the UKRR and the MS Register provide patient participants with access to their data to help them to monitor their conditions themselves. The MS Register interviewees discussed anecdotal evidence that patients were sharing results with their clinicians during consultations.

*“[W]e do something quite unusual. We actually give them data back. So, as you answer these PRO's [patient-reported outcome measures] that I've told you about. We actually give them a graph and say you're here and last time you were there, and we all look at our steps and stuff these days, so the thinking was you can monitor your MS […] they can show [their doctor] on the screen or we can send [their doctor] the link […] so we hope in the longer term this will boost participation too. [...] As well as saying oh by the way your data has gone to three publications this year, and these are them, which I’m not sure most people care about, I think it gives you a good feeling at the back end to actually you say this is for improving research in people with MS and yeah my data did make a difference there.”* (MS)

Patients’ willingness to participate was also linked to the knowledge of the individual undertaking recruitment and the need for material that clearly articulates the benefits of participating. The involvement of charities and disease organisations was also reported to foster buy-in.

*“[I]f you want to get buy in from the hospitals and the doctors that you're approaching, I think it's been really helpful for us to have the British Society for Rheumatology to help us with that because obviously all the doctors and now the nurses as well […] So the buy in from the community really comes with that kind of umbrella organization.”* (BSRBR-RA)

## Discussion

We examined six disease registries for long term conditions in the UK to explore the enablers and barriers of establishing and sustaining disease registries. Of the six registries, the CRPS is no longer actively recruiting participants while the IBD Registry announced its closure in spring 2024 “amidst NHS data landscape changes” [18]. The key challenges faced by registries were securing long-term funding and the considerable burden data collection can place on clinicians and participants, both of which were reported to be compounded by the current pressures on the NHS. Enablers included patient involvement, having appropriate infrastructure and governance structures that enable data collection to evolve over time and for linkage with other datasets, and buy-in from the community of patients and clinicians who are impacted by and/or use the data.

The UK health data landscape is currently highly fragmented, reflecting the wide range of different providers across settings, services areas and regions, and is collected by many different organisations. The registries examined sit outside the NHS-owned data flows [39]. The existence of data silos was identified as a concern by our study participants who were conscious of the very real risk registries run of duplicating existing data collection, and the additional burden data collection can place on clinicians and participants. Many of the registries have sought to reduce the burden by adopting a ‘hub-and-spoke’ model, co-defining a minimum set of measures with key stakeholders that are collected for all participants with the ability to draw in additional data either thorough linkage or primary data collection amongst all or a sub-set of participants. We heard of a number of challenges associated with linking, that have also been reported elsewhere, including timely access to data, poor interoperability between systems and governance challenges [40–43]. Having a good understanding of the existing data landscape, and suitable governance and infrastructure structures in place from the outset were seen as key enablers to supporting linkage. Storing registries in larger databanks, as illustrated by the MS Register, was also reported to improve interoperability.

The UK Government is committed to examing how data can be better managed to improve public health in England [44–46]. The Goldacre review suggests registries are an under-used resource and recommends that they need to be available for wider use through trusted research environments and that, to support this shift, registries will need to share a common computer infrastructure [44]. The Government has committed to implement Secure Data Environments (SDEs) as the default way to access NHS data [47, 48]. While the changing data landscape poses challenges to registries, as highlighted by the experience of the IBD Registry which is currently trying to identify a new home within an NHS organisation [18, 19], it could also present opportunities to overcome some of the challenges identified, if it realises its aims of facilitating improved and quicker access to data [47]. Improved interoperability between datasets would allow registries to draw on a wider set of data items and research suggests this would improve return on investment [49], potentially presenting opportunities for diseases with more limited funding resources to establish a registry. Further, simplified technology infrastructure could reduce the burden of data collection by reducing the time clinicians spend inputting data [50], releasing time not only for better patient care but also for collecting more bespoke data for registries.

The registries examined were reported to provide detailed data beyond that which is routinely collected by the healthcare system. Routine datasets in the UK inevitably lack information such as on patients’ health between their clinical encounters and patient experience such as PROMs. There are also challenges to data quality and completeness in all routine systems [41, 51, 52]. For example, the Dutch Dementia Care and Support Registry, based entirely on routinely recorded health and census data, has been found to be insufficient to meet all information needs, in particular, it lacks data on case management, quality of care and detailed information on diagnosis [53]. Interviews therefore considered that there will continue to be a need for bespoke data collection as routine data systems are not able to cover the research, clinical practice and patient needs of a registry. However, whole population disease registries based on routinely collected healthcare data have the advantage of providing more power from their large sample sizes and minimise bias related to recruitment or ascertainment. For example, the National Cancer Registration and Analysis Service (NCRAS) gathers data from a range of sources such as medical records, screening services and death certificates, covering over 300,000 cancer cases [54].

Well maintained, consent-based registries may also have advantages when there is public resistance to more extensive linkage of routine data without patient consent. For example, the failure of the English ‘care.data’ initiative shows how patient and clinician groups will oppose initiatives they suspect compromise confidentiality and fully informed consent [55–59]. As such, registries, especially for new and rare diseases, can continue to provide a potentially beneficial alternative. The key issue for the future is to establish which ‘value-added’ data items to collect and to build in sufficient flexibility to ensure new and different items can be collected without having to reconsent individual patients.

Across all the registries examined, securing long term funding has posed a major threat to their sustainability. The key cost is associated with staff time to support the day-to-day running of registry. Efforts to improve the interoperability of IT systems across the NHS and improve the data quality, discussed above, have the potential to reduce these costs. Most of the registries have drawn on multiple sources of funding, including charities related to the disease in question and many have identified commercial opportunities to raise additional funds through providing data and analysis for the pharmaceutical industry and research organisations. For example, like the BSRBR-RA, the UK's CF Registry has been working with the pharmaceutical industry to conduct long-term safety studies since 2012 to improve patient outcomes [60]. It has developed a pharmacovigilance model that requires industry partners to appoint a UK lead investigator and senior statistician to provide independent clinical guidance and registry expertise in the development of the study protocol and statistical plans, as well as conduct analyses and draft reports [61]. The partnership supports the running costs of the registry and provides an annual Registry Support Grant to each clinical centre [60]. Registries could be supported more effectively if the UK Medicines and Healthcare products Regulatory Agency and The National Institute for Health and Care Excellence (NICE) required collection of data on the effects of new medications using disease registries rather than relying exclusively on routine adverse reaction data [3]. This could enhance the independence and thus potential quality of pharmacovigilance studies in the UK as the registries are often run by charities in partnership with university researchers not necessarily associated with the trialled drug treatment. Finally, in England, the NIHR CRN was seen to have an important role by supporting research activities such as patient recruitment [62].

Despite the limited involvement of patients (and their families) when the registries were established, patient involvement is now seen as a crucial element of a successful register, and most of the registries studied have been taking steps to increase patient involvement in the design, oversight and operation functions. Other registries, have also reported that patient involvement has been particularly helpful in setting up and maintaining a disease registry [63]. However, data collection is still predominantly focused on clinical measures and only the MS Register was found to routinely collect PROMs. To capture patient-centred care it will be important to measure and report those aspects of health and wellbeing that are best described by patients themselves [64, 65]. Building in incentives for participants to remain engaged was also seen as critical. While most registries reported communicating findings and sharing publications with patients, only the MS and UKRR registries currently grant participants direct access to their data in return for their participation.

The experience of a number of the registries highlights the need to anticipate changing data needs over time and ensure that the infrastructure and governance structures allow the data collected to be used for multiple purposes and to accommodate these changing data needs. In particular, a number of registries reported challenges associated with the original patient consent obtained, which had either restricted what could be done or resulted in having to re-consent participants as their research focus and data needs shifted. Transparency in relation to the use of personal data is a requirement of the EU General Data Protection Regulation (GDPR) and the UK Data Protection Act 2018. It gives individuals the right to be informed about the data collected and its use. Our findings indicate tension between the need for transparency around how data will be used and the ability to clearly communicate how data use may change in future. These governance challenges are not unique to registries and there is a wider debate on the most appropriate models of consent for longitudinal health data collection [66–68]. Moving away from specific consent to obtaining tiered consent, dynamic consent or broad consent appears advisable so that new research questions and new ways of linking data can be employed in the future. However, the ‘best’ model of such forms of consent is debated [68–71]. Crucial for any approach will be to ensure participants are involved in developing consent procedures and adopting robust governance structures that foster trust. Any approach to consent and governance which appear rushed and do not make confidentiality a priority are also likely to be rejected by patient and clinician advocacy groups. Governance expertise must keep up with the technological pace of change for how data in the twenty-first century are collected and shared [67].

### Strengths and limitations

This study provides insights into the experience of six disease registries for long term conditions in the UK which have faced varying levels of challenge in establishing and sustaining data collection. The included registries had diverse origins, including having been established by interested clinicians and patient associations, covered diverse diseases, had different aims, set-up and funding sources, and were different sizes. Further, we included registries that have been more or less successful in securing long term funding enabling us to draw some comparisons between registries.

We identified a larger potential sample of 13 registries but were unable to secure interviews with 7 of the registries approached. Considerable efforts were made to increase participation, including contacting a named individual and sending up to three invitation emails. For two registries we were only able to identify a generic email address, and this might in part explain the non-response, additionally one individual declined due to limited capacity and a second because they did not want to formally go on record.

To inform the development of a potential long COVID registry, we focused on registries that capture individuals with long term conditions. The registries included in this study captured individuals with a clinical diagnosis, which may not be representative of registries that examine individuals based on a particular exposure or who self-identify as having a particular condition. Additionally, the focus of the interviews was on sustainability and there are other important aspects, such as the utility of registries, ensuring representativeness of the included population and how registries lead to improvements in care and outcomes, that warrant further investigation. Moreover, we included only a small number of registers (six in total), so their experiences might not be representative of registries more generally. Despite this limitation, similar themes and issues were raised across interviews suggesting that many of the enablers and challenges faced are likely to be generalisable beyond the registries examined.

## Conclusion

This study of six disease registries in the UK highlights challenges in sustaining funding and buy-in from participants over time. The changing UK data landscape and the shift to SDEs potentially poses further challenges, as highlighted by the decision of the IBD Registry to close. However, if changes, such as the arrival of SDEs, realise their potential to support easier use of linked routine and primary data, there are also likely to be opportunities for registries to reduce their costs and burden of data collection. Those involved in running registries should work together to advocate for registries within the changing NHS data landscape and make sure that they are not left behind by ensuring that their registry remains interoperable with other data sources and systems.

Just as surveillance systems for infectious diseases have a crucial role to play in the public health identification of, and response to, outbreaks and pandemics, so too the contribution of registries for non-communicable diseases and infections with long-term adverse impacts such as COVID-19 should be recognised as an important plank in the biomedical and health services research infrastructure, benefiting basic research, clinical management and patient engagement. Registries will continue to provide valuable insight above and beyond routine data for the foreseeable future, and our findings identify enablers to establishing and sustaining registries that provide insights for those considering developing disease registries. Table 3 outlines the key considerations for establishing a registry. Key among these is ensuring inclusivity by engaging diverse stakeholders in the planning and running of the registry, identifying the core set of data measures needed to address the registry’s priorities, and establishing appropriate consent processes that allow the data collected to change over time and to link to other data sets. Finally, registries should explore different sources of funding including commercial opportunities.

**Table 3:** Key considerations for establishing a registry

1. **Involve patients at every stage:** develop formal processes to ensure active involvement of patients (and their families/carers) in all aspects of the registry from the initial design phase, including priority setting, to oversight and operational functions such as representation on data approval committees and governance boards, through to dissemination
2. **Include a diverse set of stakeholders:** establish an inclusive steering committee to ensure membership reflects the diversity of individuals who will be impacted by and/or benefit from the registry’s activities. This will include but is not limited to patients (and their families/carers), representatives of associated disease charities, clinicians (including those outside of the disease speciality), researchers (including those from outside the UK), data managers (including individuals with an awareness of the wider UK data landscape) etc
3. **Collect a core data set for all participants:** maximise efficiency by identifying a core data set to reduce the burden on patients and clinicians. Work with individuals who will be impacted by and/or benefit from the registry to define the registry’s purpose and the data of most value needed to address these questions. Map the wider data landscape to ensure only data that are not already routinely collected are requested from participants
4. **Ensure the data system is flexible and interoperable with the wider data landscape:** build in mechanisms that allow the data collected to be easily expanded. Collecting identifiable information (NHS number, date of birth, postcode) allows linkage with existing data sets and/or establishing consent procedures that allow participants to be easily contacted to participate in additional data collection or studies helps to enable such linkage
5. **Anticipate changing data needs over time:** set up transparent consent and permission processes that allow (reasonable) changes to data collection and use, and the potential to link to additional (unknown) data sources at a future date in response to changing priorities without having to reconsent participants. This depends on transparency around data use and research finding to build long term trusts
6. **Identify financial opportunities to sustain the registry’s activities for the long term:** these might include commercial opportunities, where consistent with the aims of the registry. Where feasible, it is a priority to embed the registry within the NHS research system, principally by inclusion on the NIHR CRN Portfolio

## Data Availability

The datasets generated and/or analysed during the current study are not publicly available as the data contain potentially sensitive participant information and consent was only sought to share anonymous quotations specifically for the purposes of this study but are available from the corresponding author on reasonable request.

## Abbreviations

BSRBR-RA: British Society for Rheumatology Biologics Register for Rheumatoid Arthritis
CF: Cystic Fibrosis
CFS: Chronic Fatigue Syndrome
CRN: (NIHR’s) Clinical Research Network
CRPS: Complex Regional Pain Syndrome
GDPR: General Data Protection Regulation
IBD: Inflammatory Bowel Disease
JSLE: Juvenile-onset Systemic Lupus Erythematosus
ME: Myalgic Encephalomyelitis
MND: Motor Neuron Disease
MS: Multiple Sclerosis
NHS: National Health Service
NICE: National Institute for Health and Care Excellence
NIHR: National Institute for Health and Care Research
PROMs: Patient Reported Outcome Measures
SAIL: Secure Anonymised Information Linkage
SDE: Secure Data Environment
TNF: Tumour Necrosis Factor
